# Supplementary material for: Identification of photoperiod-induced specific miRNAs in the adrenal glands of Sunite sheep (Ovis aries)
Source: Front Vet Sci. 2022 Jul 22;9:888207. doi: 10.3389/fvets.2022.888207 (PMC9354845; doi:10.3389/fvets.2022.888207)
Supplement: Supplementary file 1 [file Data_Sheet_1.ZIP › Supplementary file/miRDeep2 file.zip!/miRDeep2 file/Know_Structure/LP42A2/oar-mir-22.pdf]

miRBase precursor : oar-mir-22
Total read count : 114676
oar-miR-22-3p read count : 110464
remaining reads : 4212

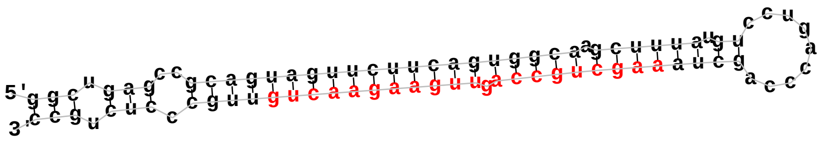

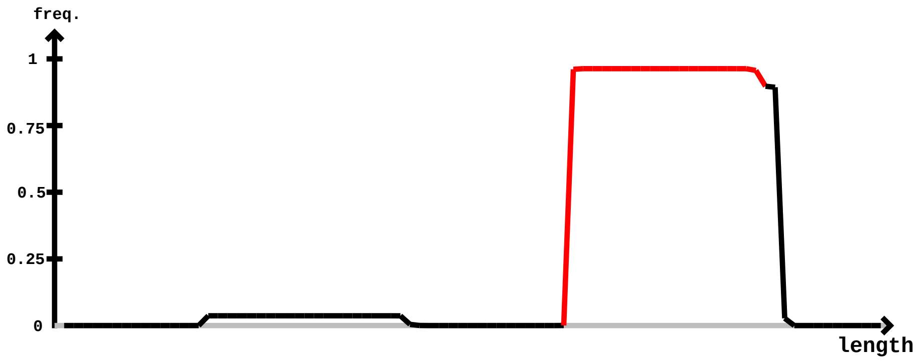

oar-miR-22-3p

5'- ggcugagccgcaguaguucuucaguggcaagcuuuauguccugacccagcuaaagcugccaguugaagaacuguugcccucugcc -3'	exp		
(((.(((..((((((((((((((((((((.((((((.((.........))))))))))))).))))))))))))))).))).)))	reads	mm	sample
............uaguucuucaguggcaagcuuu..	16	0	seq
.............aguucuucaguggcaagcu..	5	0	seq
.............aguucuucaguggcaagcuu...	23	0	seq
.............aguucuucaguggcaagcuuu..	3646	0	seq
.............aguucuucaguggcaagcuuua...	488	0	seq
.............aguucuucaguggcaagcuuuau..	29	0	seq
..............guucuucaguggcaagcuuu..	3	0	seq
..............guucuucaguggcaagcuuua...	1	0	seq
....cuaaagcugccaguugaagaacu.............	1	0	seq
....aaagcugccaguugaagaacu.............	15	0	seq
....aaagcugccaguugaagaacug............	1	0	seq
....aaagcugccaguugaagaacugu...........	13	0	seq
....aagcugccaguugaaga................	4	0	seq
....aagcugccaguugaagaa...............	16	0	seq
....aagcugccaguugaagaac..............	754	0	seq
....aagcugccaguugaagaacu.............	6743	0	seq
....aagcugccaguuCaagaacu.............	1	1	seq
....aagcugccaguugaagaacug............	440	0	seq
....aagcugccaguugaagaacugu...........	99102	0	seq
....aagcugccaguugaagaacuguu..........	3096	0	seq
....aagcugccaguugaagaacuguug.........	3	0	seq
....agcugccaguugaagaacu.............	18	0	seq
....agcugccaguugaagaacugu...........	231	0	seq
....agcugccaguugaagaacuguu..........	26	0	seq
....gcugccaguugaagaacugu...........	1	0	seq
